# Development of Versatile Vectors for Heterologous Expression in *Bacillus*

**DOI:** 10.3390/microorganisms6020051

**Published:** 2018-06-07

**Authors:** Øivind Larsen, Gro Elin Kjæreng Bjerga

**Affiliations:** Centre for Applied Biotechnology, Uni Research AS, Thormøhlens gt. 55, N-5006 Bergen, Norway; oivind.larsen@uni.no

**Keywords:** cloning, recombinant DNA technology, *ccd*B, subtilisin, *Bacillus*

## Abstract

The discovery of new enzymes for industrial application relies on a robust discovery pipeline. Such a pipeline should facilitate efficient molecular cloning, recombinant expression and functional screening procedures. Previously, we have developed a vector set for heterologous expression in *Escherichia coli*. Here, we supplement the catalogue with vectors for expression in *Bacillus.* The vectors are made compatible with a versatile cloning procedure based on type IIS restriction enzymes and T4 DNA ligase, and encompass an effective counter-selection procedure and complement the set of vectors with options for secreted expression. We validate the system with expression of recombinant subtilisins, which are generally challenging to express in a heterologous system. The complementarity of the *E. coli* and *Bacillus* systems allows rapid switching between the two commonly used hosts without comprehensive intermediate cloning steps. The vectors described are not limited to the expression of certain enzymes, but could also be applied for the expression of other enzymes for more generalized enzyme discovery or development.

## 1. Introduction

Due to their wide application range, the discovery and development of proteases have a great economic potential. An enzyme discovery pipeline should facilitate efficient molecular cloning, recombinant expression and functional screening procedures [1,2], but often requires adaptation to the enzyme of interest. Serine proteases constitute about a third of known proteolytic enzymes, including the subtilisin family [3]. The subtilisins derived their name from *Bacillus subtilis*, from which the enzyme was first isolated [4], but they are widespread, being found in bacteria, archaea, viruses and eukaryotes [5]. Certain members of the subtilisin family, such as the extracellular subtilisin proteases (ESPs), have been extensively researched and used in the detergent, leather and food industries [6]. Endogenous ESPs are produced as inactive precursor proteins consisting of a leader sequence [7] that directs their export, a pro-sequence required for folding [8,9] and the catalytic domain. The latter classifies as a Peptidase S8 (PF00082) domain in the Pfam classification [10]. The leader sequence in ESPs is a typical secretory (Sec) sequence [11] directing export of the enzyme using the Sec-dependent pathway, which is the most common pathway for secretion [11]. The pro-sequence has a dual role and acts as both an inhibitor and as a molecular chaperone that guide correct folding of the enzyme [12,13,14], and is removed by autoproteolysis. The autoproteolytic maturation poses a challenge for heterologous production. It has, however, been shown that ESPs eliminated for the leader sequence but retaining the pro-domain can be produced in the commonly used host *Escherichia coli* [12,15,16]. Furthermore, a comprehensive pipeline has been generated that facilitates parallel, directional cloning of genes to a vector set compatible with recombinant expression in *E. coli* [16]. The established system has proven successful for expression of more than 100 enzymes, albeit not limited to proteases [17]. However, metagenomic discovery efforts, where *E. coli* is frequently used as a host, often suffer from a low number of positive clones [18] despite the relatively high number of subtilisins per genome and their wide representation in organisms [19]. The difficulties in expressing subtilisins and obtaining them in an active form are likely due to the complex maturation and intrinsic proteolytic activity if unregulated. However, yields for downstream characterization and application are often insufficient in *E. coli*. Secretion of enzymes from the host is an attractive and cost-efficient production system due to limited needs for cell processing and elaborate purifications. *Bacillus subtilis* is such an attractive host because of its large capacity to export enzymes. Besides, it is non-pathogenic and is generally regarded as safe (GRAS) by the US Food and Drug Administration, which make it a suitable host for enzymes that will be used in food applications. However, regular *B. subtilis* species produce a number of extracellular proteases that can potentially be detrimental for heterologous expression. Moreover, native proteases pose a challenge for the assessment of recombinant proteases as they provide a background activity in biochemical assays. For this purpose, protease-deficient strains, such as the WB800-derivatives [20], are preferred.

To facilitate efficient molecular cloning of a large number of genes in parallel, a range of assembly cloning techniques have recently been developed based on type IIS restriction enzymes and T4 DNA ligase [21,22,23]. With several of these methods a counter-selection approach using the coupled cell division B gene (*ccd*B) gene is used [23]. The negative selection is based on the presence of the *ccd*B-gene in the cloning region of the vector which, upon sub-cloning, is replaced with the gene of interest. This promotes the emergence of positive clones, as negative clones will express a cytotoxic protein encoded by the *ccd*B-gene that cause gyrase-mediated chromosomal damage and ultimately cell death [24]. For high-throughput cloning, counter selection is identified as particularly useful to limited elaborate screening for positive clones. This has previously shown great advantages for recombinational cloning [25,26].

In this study, we have developed three vectors for heterologous expression in *Bacillus*, here explored in *B. subtilis* WB800N, and validated these by production of both intracellular (green fluorescent protein) and extracellular (subtilisin) proteins. The vectors are compatible with a versatile cloning regime based on fragment exchange (FX), encompassing an effective counter-selection procedure and implementing secreted expression. The *Bacillus* system complement the previously developed system for *E. coli* [16], and allows rapid switching between two commonly used heterologous host systems without comprehensive intermediate cloning steps.

## 2. Materials and Methods

### 2.1. Construction of Fragment Exchange (FX)-Compatible Vectors for Bacillus Expression

Three vectors were designed for FX-compatible cloning [23] and recombinant expression in *Bacillus* (Table S1). The p17-vector allows intracellular or secreted expression depending on the absence or presence of native leader sequences in the sub-cloned gene, respectively. The p18- and p19-vectors contain leader sequences reported to efficiently direct export of the recombinant proteins [27]. All vectors contain C-terminal hexahistidine tags. As starting templates, two vectors that had successfully been used in *Bacillus*, pSP_LipA_-hp and pSP_YocH_-hp (MoBiTec, Table S1), were used [27]. To enable FX compatibility, a counter selection cassette containing the *ccd*B gene and a chloramphenicol-resistance gene *(cam*R) flanked by *Sap*I restriction sites were added to the cloning region of the templates using a megapriming polymerase chain reaction (PCR) cloning method [28]. The cassette was amplified from the p1 vector [16,23] using cloning primers listed in Table S2 and Phusion polymerase (New England Biolabs, Hitchin, UK) protocol and purified with the QIAquick PCR purification kit (QIAGEN, Valencia, CA, US). The cassette was inserted to *Bam*HI linearized pSP_LipA_-hp and pSP_YocH_-hp vectors, and used as templates in the linear plasmid amplification reaction as described elsewhere [16,28,29,30]. To identify whether product formation occurred, the reaction mixture was screened by PCR using primers flanking the insertion site (Table S2). To remove template DNA, the PCR products were digested with 10 U *Dpn*I (New England Biolabs, Hitchin, UK) and transformed to *E. coli* MC1061 cells (Table S1). Plasmids were isolated using the NucleoSpin plasmid purification kit (MACHEREY-NAGEL GmbH & Co, Düren, Germany). Sanger sequencing was used to confirm correct cloning of all vectors. Plasmids and strains used in this study are detailed in Table S1.

### 2.2. Sub-Cloning of gfp and apr Genes to the FX-Compatible Bacillus Vectors

The *gfp* gene (coding for residues 2–247 of green fluorescent protein, GFP) was sub-cloned from the *B. megaterium* optimized pSSBm85 plasmid [27] into the pINITIAL cloning vector [23] and verified by sequencing. Codon-optimized *apr* genes (Genscript, Piscataway, NJ, USA) encoding subtilisins from *B. licheniformis* DSM13, *B. paralicheniformis* ATCC 9945A, *B. subtilis* subsp. *subtilis* str. 168 and *B. amyloliquefaciens* (GenBank IDs: AAU40017, AGN35600, CAB12870 and AAB05345, respectively, herein termed: B13, B9945, BSU and BAM) [16] served as templates for PCRs using Phusion polymerase (NEB). Genes were integrated to the pINITIAL cloning vector [16,23] and sequenced to confirm correct cloning. Sub-cloning of genes from pINITIAL to the FX-compatible *Bacillus* expression vectors were carried out as described previously [16,23]. Empty vectors were generated by replacing the *ccd*B-*cam*R cassette with a GSGSGS (GS) linker to allow their propagation in *E. coli* MC1061 cells, as described previously [16], and use as background controls in experiments.

### 2.3. Transformation of Bacillus subtilis by Natural Competence

The *Bacillus subtilis* WB800N strain [20], utilized for heterologous expression, was transformation-based using a protocol developed by Anagnostopoulus and Spizizen [31]. A single colony of *B. subtilis* WB800N from an lysogeny broth (LB) agar plate (1% (*w*/*v*) tryptone, 0.5% (*w*/*v*) yeast extract, 1% (*w*/*v*) NaCl, 1.5% (*w*/*v*) agar-agar) was inoculated in freshly prepared minimal medium (60 mM K_2_HPO_4_, 40 mM KH_2_PO_4_, 3 mM trisodium citrate, 20 mM potassium-l-glutamate, 3 mM MgSO_4_, 1% glucose, 20 µg/mL l-tryptophane, 0.1% casamino acids) and grown for 16–20 h at 37 °C and 250 rpm. The culture was diluted to an optical density at 600 nm (OD_600_) of 0.2 with minimal medium, and grown for 4 h at the conditions given above. Cells were harvested, diluted 10-fold and distributed in 1 mL aliquots for individual transformations. Typically 0.5–1.0 µg plasmid DNA was added to cells, and incubated for 6 h at the conditions given above. Cells were harvested and spread on LB agar plates supplemented with 10 µg/mL tetracycline. Colonies were checked for the presence of correct plasmid by PCR.

### 2.4. Heterologous Expression of Green Fluorescent Protein (GFP) and Subtilisins in Bacillus subtilis

1 mL LB media (1% (*w*/*v*) tryptone, 0.5% (*w*/*v*) yeast extract, 1% (*w*/*v*) NaCl) containing 10 µg/mL tetracycline was added to deep 24-well plates, and inoculated with single colonies from agar plates and grown at 37 °C and 750 rpm. 100 µL pre-culture was used to inoculate 4 mL 2YT media (1.6% (*w*/*v*) tryptone, 1% (*w*/*v*) yeast extract and 0.5% (*w*/*v*) NaCl) with 10 µg/mL tetracycline. Cells were incubated for 3–4 h to reach log phase, prior to the induction of expression with 0.1% (*v*/*v*) d-xylose (Sigma-Aldrich, St. Louis, MO, USA) for 16–20 h at 20 °C (GFP) or 37 °C (proteases) and 750 rpm. Cells were harvested using an Allegra X-12R benchtop centrifuge (Beckman Coulter, Brea, CA, USA) at 4750 rpm for 15 min. Proteins in 1 mL supernatants were precipitated with trichloroacetic acid (TCA; 10% final concentration) for 1 h at 37 °C, washed twice in 500 µL acetone, and resuspended in 40 µL 1× Laemmli sample buffer (Bio-Rad Laboratories, Hercules, CA, USA) for sodium dodecyl sulfate polyacrylamide gel electrophoresis (SDS-PAGE) analysis [32]. Cells were resuspended in 1 mL 8.5 N lysis buffer (50 mM Tris HCl pH 8.5, 50 mM NaCl, 0.25 mg/mL lysozyme, 10% (*v*/*v*) glycerol), and lysed by ultrasound as previously described [16]. Cleared lysates (soluble fraction) and pellets (insoluble fraction) were analyzed by SDS-PAGE [32].

### 2.5. GFP Fluorescence Measurement

Fluorescence from 100 µL cell cultures containing GFP was measured with excitation at 485 nm and emission at 520 nm using a Sense microplate reader (Hidex, Turku, Finland).

### 2.6. Detection of Recombinant Subtilisins by Immunoblotting

Proteins from supernatants and cleared lysates were analyzed by SDS-PAGE, and transferred onto a nitrocellulose membrane [33] using the Trans-Blot Turbo (Bio-Rad Laboratories) transfer system. A mouse monoclonal anti-polyhistidine antibody (Cat. No. H1029, Sigma-Aldrich) was used to identify expression of recombinant subtilisins with C-terminal histidine affinity tags. The primary antibodies were detected with a secondary rabbit horseradish peroxidase linked mouse IgG (NA931V, GE Healthcare, Little Chalfont, UK). The HRP-reaction was developed with the Clarity Western ECL Substrate (Bio-Rad Laboratories), and imaged in the Chemi-Doc gel imager (Bio-Rad Laboratories).

### 2.7. Subtilisin Activity Assays

Proteolytic activity was assessed using EnzChek™ Protease Assay Kit (Thermo Fisher Scientific, Waltham, MA, USA). 10 µg/mL BODIPY FL casein was prepared by resuspending the substrate in 50 mM Tris HCl pH 8.5 (at RT) and 50 mM NaCl. 12.5 µL of BODIPY-FL casein was used per reaction, with 10 µL supernatant from expression in assay buffer I (50 mM NaCl, 50 mM TrisHCl pH 8.5 at room temperature) in a final volume of 100 µL in MicroFluor 1 plates (Thermo Fisher Scientific). Samples were incubated at 37 °C for 1 h unless otherwise stated, and fluorescence was read. Fluorescence was measured at excitation 485 nm and emission 520 nm using the Hidex Sense microplate reader. Routinely, Alcalase™ 2.4L (Sigma-Aldrich) was used at a dilution 1:10,000 in 8.5 N lysis buffer.

Temperature activity profiles for recombinant subtilisins were determined from 15 µL supernatants in assay buffer II (80 mM NaCl, 80 mM TrisHCl buffer pH 8.2 at the experimental temperatures) and 3.3 µM *N*-succinyl-l-Ala-l-Ala-l-Pro-l-Ala (AAPA) *p*-nitroanilide (Bachem, Weil am Rhein, Germany; resuspended in dimethyl sulfoxide) in a total volume of 150 µL. The assay was carried out for 10 min at the relevant temperatures, and stopped by addition of acetic acid at a final concentration of 80 mM. 150 µL of the reaction volume was then transferred to a microplate for absorbance reading at 405 nm in the Hidex Sense microplate reader. pH activity profiles were determined for 15 µL supernatant as above, but using either 80 mM citrate buffer pH 3.0–6.0, 80 mM Tris-HCl buffer pH 7.3, 8.1, 9.0 and 80 mM glycine buffer pH 10.0 Reaction was run at 37 °C and increase in absorbance at 405 nm was monitored. In both experiments, the obtained absorbance data were background subtracted (backgrounds are supernatants from cells expressing the GS-linker from vectors), and presented in relative activity (% of maximum activity). The measurements were carried out with two technical replicates in three biological replicates. GraphPad Prism 7 (GraphPad Software, La Jolla, CA, USA) was used to prepare plots and to perform statistical analyses.

## 3. Results

### 3.1. Preparation of FX-Compatible Bacillus Vectors

**Figure 1:**
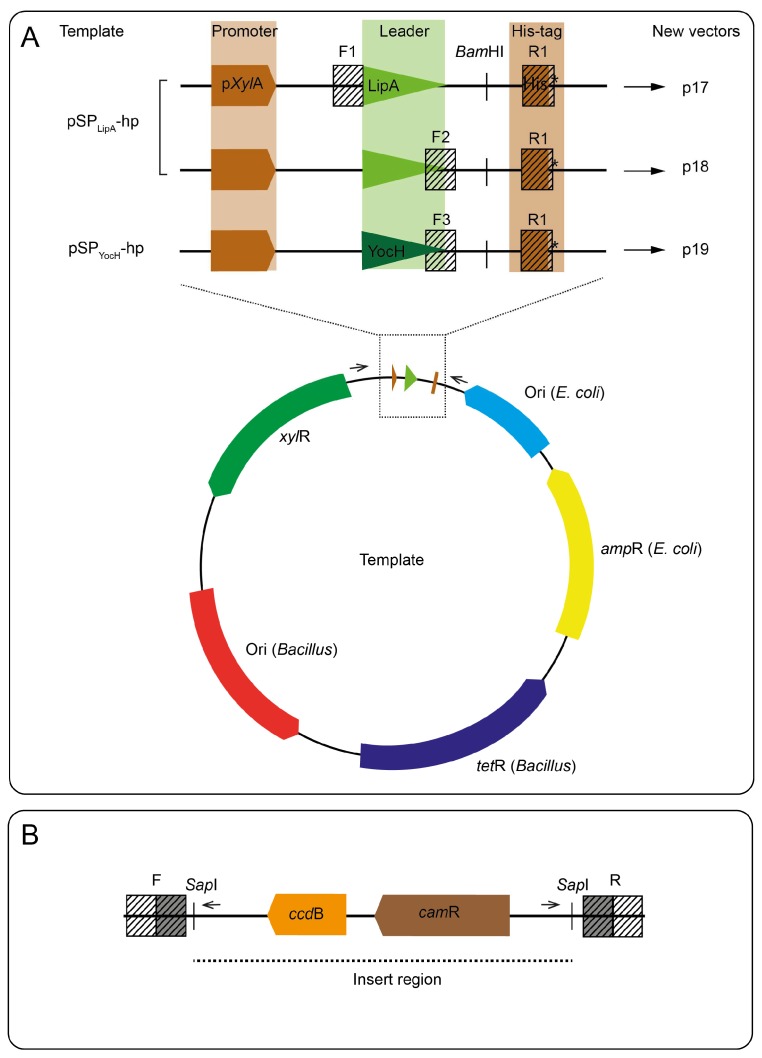
Design of fragment exchange (FX) compatible *Bacillus* expression vectors. (**A**) The vector templates, pSP_LipA_-hp and pSP_YocH_-hp, have a common backbone consisting of two origins of replication (one for *E. coli* and one for *Bacillus*), two antibiotic resistance genes (for ampicillin selection in *E. coli* and tetracycline selection in *Bacillus*) and the *Bacillus* gene encoding the regulatory protein, XylR. The highlighted regions (upper panel) consist of the *Bacillus* p*Xyl*A promoter, two different leader sequences (LipA and YocH leader sequences, respectively), and C-terminal histidine affinity tags (His-tag) for downstream purification of the recombinant proteins. The hatched boxes (F- and R-sites) indicate the vector-specific regions used for insertion of the *ccd*B-*cam*R cassette. *Bam*HI indicates the restriction site used for linearization, and arrows indicate position and direction of screening primers; (**B**) The region inserted between the F-sites and the R-site of the pSP_LipA_-hp and pSP_YocH_-hp templates to generate the three vectors p17, p18 and p19, as shown in **A**, by whole plasmid amplification. The region contains the *ccd*B and *cam*R genes, and flanking *Sap*I sites. Hatched boxes marked F and R are specific to each vector design and consists of two parts; it has an overlapping region for gene-specific amplification (filled boxes), and extensions that are complementary to the insertion site (open boxes) in the templates (F = either F1, F2 or F3; R = R1, as indicated in **A**). The insert region designates the region in the final vectors that is replaced upon sub-cloning of recombinant genes, and arrows show position and direction of sequencing primers.

To facilitate screening of industrially relevant enzymes, such as subtilisins, efficient systems for high-throughput cloning and expression is essential. Here, we have developed three vectors for heterologous expression in *Bacillus* [20], named p17, p18, and p19, by adapting existing *Bacillus*-compatible plasmids to the FX-cloning principle (Figure 1). The *ccd*B-*cam*R cassette was inserted to the pSP_LipA_-hp and pSP_YocH_-hp plasmids by linear amplification using a PCR product containing the cassette sequence as a megaprimer [28]. To confirm product formation before continuing with transformation, the PCR reaction itself was screened using primers flanking the insertion site. In these cases, the screening process identified a mix of both templates and products. During *Dpn*I-digestions, the templates were removed, and positive clones selected. The three vectors allow intracellular as well as secreted expression. The p17 vector does not harbor a leader sequence, thus allowing exploitation of the putative native leader sequence from the enzyme of interest. The p18 and p19 vectors harbor known LipA and YocH leader sequences, respectively, which are known to facilitate export in *Bacillus* [34].

### 3.2. Expression of GFP in Bacillus subtilis

**Figure 2:**
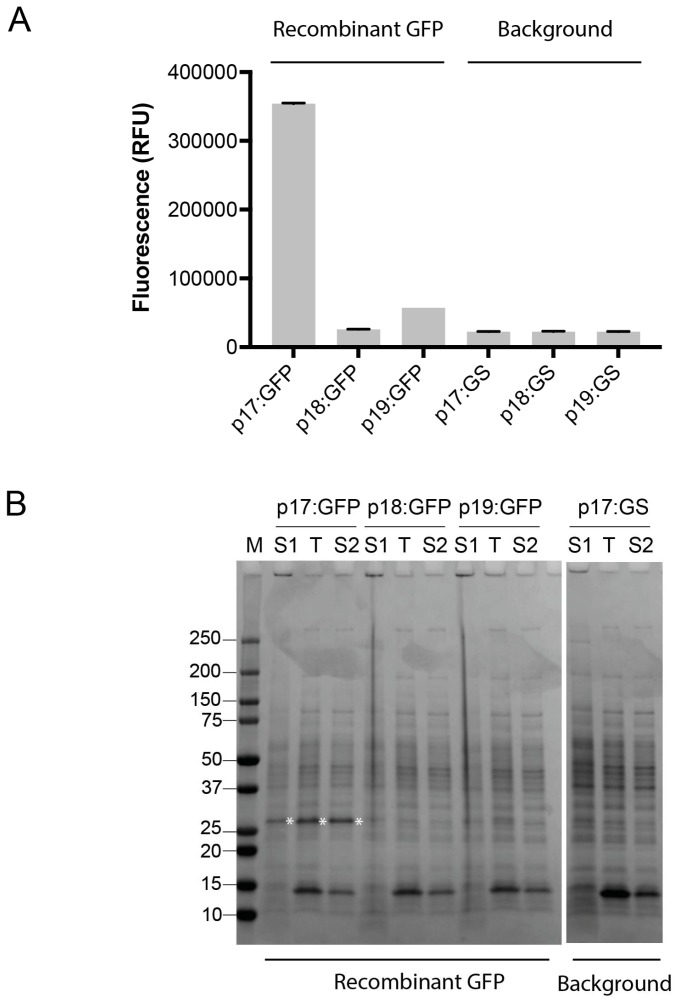
Recombinant expression of green fluorescent protein (GFP) in *B. subtilis* WB800N. (**A**) GFP expression from the p17 to p19 vectors was assessed by fluorescence measurements taken from the expression cultures. Fluorescence was compared to background controls (in which a GSGSGS-linker (GS) is replacing GFP in the vectors). Data is shown from one representative experiment, and error bars represent deviation in two technical replicates; (**B**) Sodium dodecyl sulfate polyacrylamide gel electrophoresis (SDS-PAGE) analysis of fractions from expression of GFP from the p17 to p19 vectors. S1, supernatant fraction; T, total protein in the cellular fraction; S2, soluble fraction of protein in cells. M, Precision Plus protein standard (Bio-Rad Laboratories).

To validate the three vectors, the *gfp* gene encoding green fluorescent protein was used for simple fluorescence-based monitoring of recombinant protein production. The gene encoding GFP was sub-cloned from the *B. megaterium* optimized pSSBm85 plasmid [27] into the pINITIAL cloning vector, and sub-cloned into the *Bacillus* vectors. Expression was achieved by xylose induction. Fluorescence measurements were taken from the cultures directly. GFP, which does not contain a leader sequence, was found to fluoresce in the p17-based cultures (Figure 2A), indicating soluble expression of an intracellular GFP. SDS-PAGE analysis of fractions from expression revealed that recombinant GFP was indeed expressed from p17, and primarily in the cellular fraction (Figure 2B). Export was low or not obtained by adding the LipA or YocH leader sequences in front of the *gfp* gene (Figure 2).

### 3.3. Validation of Vectors by Expression of Active Recombinant Subtilisin Proteins in Bacillus subtilis

**Figure 3:**
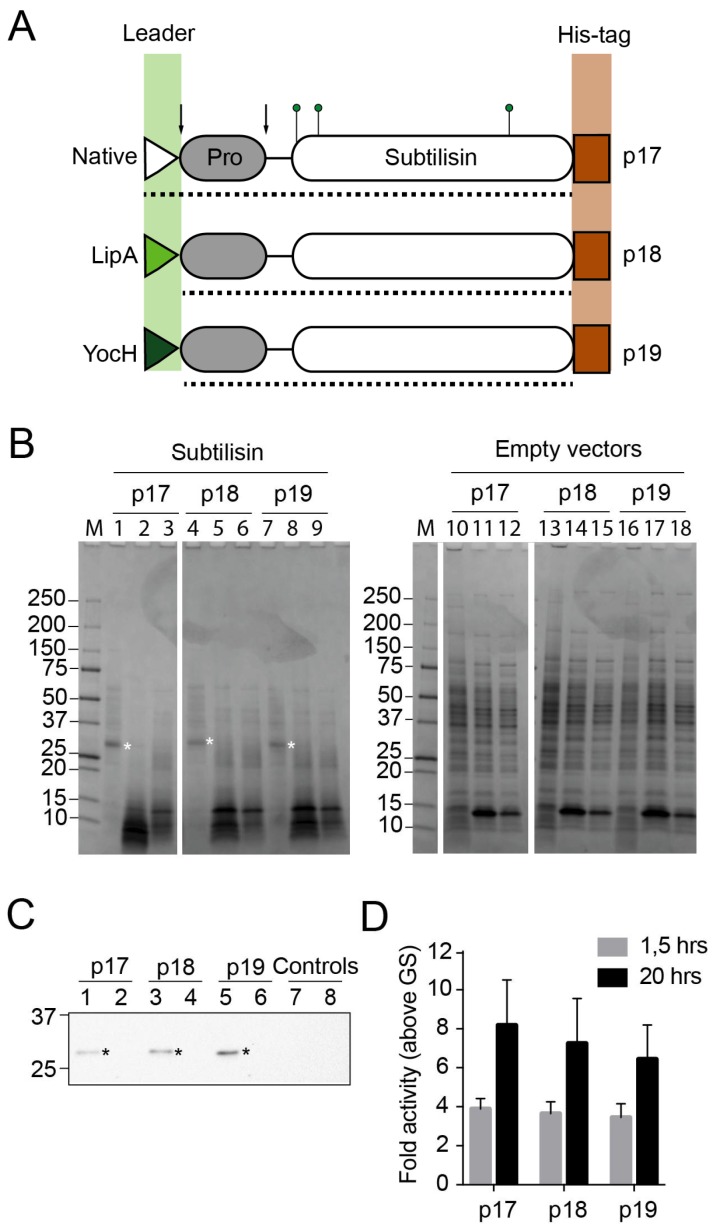
Recombinant expression and activity of subtilisins from FX-compatible *Bacillus* vectors. (**A**) Cartoon of recombinant subtilisin versions expressed from p17, p18 and p19 vectors, showing the leader sequence (triangles), pro-domain (grey boxes), catalytic domain (white boxes) and C-terminal histidine affinity tag (brown rectangles). Green pins point to residues involved in catalysis (catalytic triad). Dotted line shows the region of the protein encoded by the sub-cloned *Bacillus licheniformis* DSM13 *apr* gene. In the p18 and p19 vectors, the vector-encoded LipA or YocH leader sequences (green triangles) replace the native leader sequence (white triangle) in subtilisin, respectively. Arrows show processing sites for leader sequence removal and pro-domain cleavage. Illustration is drawn to scale; (**B**) SDS-PAGE analysis of recombinant subtilisins expressed in *B. subtilis* WB800N (left panel) and empty vector controls (right panel). Lanes 1, 4 and 7 show supernatant fractions from cultures expressing subtilisin from p17, p18 and p19, respectively. Lanes 2, 5 and 8 contains soluble fractions from cleared cell lysates. Lanes 3, 6 and 9 contains insoluble fractions from cell lysates. Lanes 10–18 are organized accordingly, but contain fractions from cultures with empty vectors (subtilisin is replaced with the GS-linker). M, Precision Plus protein standard are shown to the left of both panels; (**C**) Immunoblot of recombinant subtilisin using antibodies against the C-terminal his-tag. Supernatants and soluble fractions of subtilisin expression from p17 (lanes 1–2), p18 (lanes 3–4) and p19 vectors (lanes 5–6) in *B. subtilis* WB800N, respectively. As control, fractions from expression of empty p17 are shown in lanes 7–8. Asterisks indicate bands that correspond to the expected mass of matured subtilisins (28 kDa). Two bands from the Precision Plus protein standard are shown to the left; (**D**) The supernatants of *B. subtilis* WB800N expression cultures containing the subtilisin versions (expressed from p17, p18 and p19 vectors, respectively) were screened for proteolytic activity against BODIPY-conjugated casein for 1.5 and 20 h. Fluorescence values were made relative to empty vector controls (GS-linker), and errors show deviation in three biological replicates.

To assess the capacity for secreted expression, the *apr* gene encoding extracellular subtilisin proteases from *B. licheniformis* DSM13 was used (Figure 3A). The entire *apr* gene, encoding subtilisin with the native leader sequence (residues 1–379), was sub-cloned from pINITIAL to p17. The truncated gene encoding pro-subtilisin (residues 30–379) without the leader sequence, was sub-cloned to p18 and p19. In the latter two constructs, the native leader sequences of subtilisin were replaced with the LipA and YocH leader sequences encoded by the vectors, respectively. SDS-PAGE analysis from heterologous expression showed that the recombinant subtilisin was secreted to the media after induction and expression from all three vectors (Figure 3B). Recombinant subtilisin was not identified in the cellular fractions. Immunoblots using anti-his antibodies against the C-terminal his-tags of the recombinant subtilisins supported these findings (Figure 3C). To measure the activity of the recombinant subtilisin, we used an in vitro casein-based protease assay. The activity detected in the supernatant fractions was 3–11 times higher than in control samples depending on conditions (Figure 3D), and all versions of the recombinant subtilisin showed comparable activity.

**Figure 4:**
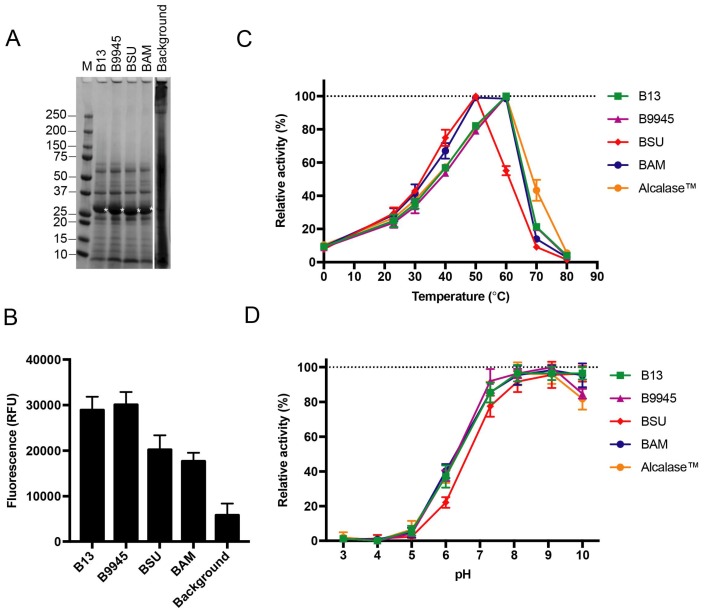
Four different subtilisin proteins expressed from FX-compatible *Bacillus* vectors. (**A**) A representative SDS-PAGE showing the supernatant fraction of the *B. subtilis* WB800N host after recombinant expression of native subtilisins from *B. licheniformis* DSM13 (B13), *B. paralicheniformis* ATCC 9945A (B9945), *B. subtilis* subsp. *subtilis* str. 168 (BSU) and *B. amyloliquefaciens* (BAM). Asterisks indicate recombinant enzymes (matured subtilisins expected mass is 28 kDa). Background represents expression of the empty vector; (**B**) Supernatants in A were screened for proteolytic activity against BODIPY-conjugated casein. Error bars show deviation in three biological replicates; (**C**) Temperature activity profiles of recombinant subtilisins (B13, green; B9945, magenta; BSU, red; BAM, blue) screened for proteolytic activity against the chromogenic succinyl-AAPA peptide, and compared to commercial Alcalase™ 2.4L (orange). Error bars show deviation in three biological replicates; (**D**) pH activity profiles of recombinant subtilisins screened against the peptide in C. Error bars show deviation in three biological replicates.

We performed a comparative study on four subtilisins, including the one encoded by the *B. licheniformis* DSM13 *apr* gene and three other *apr* genes from *B. paralicheniformis* ATCC 9945A, *B. subtilis* subsp. *subtilis* str. 168 and *B. amyloliquefaciens* previously used in a similar mini screen [16]. The three additional full-length *apr* genes with native leader sequences were sub-cloned into the p17 vector. Each of the four subtilisins was expressed, and the supernatants were tested for proteolytic activity in the in vitro casein assay (Figure 4). All four subtilisins were exported from the host cell and identified as soluble enzymes in the supernatant (Figure 4A), and found to be active (*p*-value < 0.00001; Figure 4B).

### 3.4. Initial Characterisation of Four Different Subtilisin Proteins

To demonstrate the functionality of the recombinant enzymes, we characterized their activity profiles. The temperature activity profiles of the recombinant subtilisins were assessed with a synthetic peptide, succinyl-l-Ala-l-Ala-l-Pro-l-Ala (AAPA), commonly used to address substrate specificity [35,36]. The two recombinant subtilisins from *B. licheniformis* and *B. paralicheniformis* and the commercial enzyme formula Alcalase™ 2.4L, that originates from *B. licheniformis*, peaked at 60 °C (Figure 4C). The *B. subtilis* homolog had an optimal temperature at 50 °C, and only 55% of its activity remained at 60 °C. The *B. amyloliquefaciens* homolog had a broader temperature profile, with optimal temperature at 50 °C, which remained largely unchanged at 60 °C. All of the enzymes had lost most of the activity at 80 °C. The recombinant subtilisins were all active at pH 5.0 and above, where a peak of activity was reached at pH 8.0–10.0 for all enzymes (Figure 4D). Subtilisins from *B. subtilis, B. licheniformis* and *B. amyloliquefaciens* are, however, more active at pH 10 than the other enzymes (*p*-value < 0.00001).

The two recombinant subtilisin homologs from *B. licheniformis and B. paralicheniformis* appeared to share similar activity profiles, both in terms of temperature and pH preferences. They also aligned well with the activity profile of the commercial Alcalase™ 2.4L (Figure 4C,D).

## 4. Discussion

The *Bacillus* fragment exchange (FX) vector system was designed to complement the intracellular *E. coli* system [16] by facilitating secreted expression. These vectors would be useful for naturally secreted proteins, but also for exploring the export of proteins that otherwise would be expressed in the intracellular compartment.

The pSP_LipA_-hp and pSP_YocH_-hp plasmids, initially designed for mediating high-yield production in *B. megaterium* [27], were used as templates to design FX-cloning compatible vectors for heterologous expression. To develop the vectors, we used an approach exploring linear amplification that uses the insert DNA fragment as a megaprimer for polymerase-mediated elongation [28]. The method has previously been used to design new vectors [30,37,38]. Commonly, such long-range amplifications are carried out on circular templates (plasmids), but we found that *Bam*HI-linearized templates gave higher product formation than circular templates as assessed by agarose gel electrophoresis [39]. Evaluation of this as a general optimization procedure for large and difficult plasmid amplifications is outside the scope of this study.

The new vectors facilitate expression in the presence of xylose due to a strong inducible pXylA promoter [38], which is repressed in the absence of inducer by the XylR repressor (Figure 1). The promoter has previously been shown to drive heterologous expression in *B. subtilis* [40]. In a previous screen of leader sequences applicable for high-yield production, the YocH and LipA were shown to mediate efficient secretion of a thermophilic ester-hydrolase into the growth medium in *B. megaterium* [27]. The LipA and YocH leader sequences originate from an esterase and a cell wall binding protein in *B. megaterium* [27,41], respectively, but are conserved in *B. subtilis* [11]. As addressed in previous reports, the YocH and LipA leader sequence show high similarity with the consensus sequence for type I leader sequences directing Sec-dependent export. We were, therefore, confident that the vector systems would be suitable for heterologous expression not only in *B. megaterium*, but also in *B. subtilis* WB800N.

The system was initially validated with expression of the GFP from the p17 vector (Figure 2). Expression trials with p18 and p19 vectors were not giving traceable amounts of GFP (Figure 2), which is likely explained by the fact that GFP is not directed for export using the Sec pathway [42]. To validate Sec-dependent export of proteins, subtilisin from *B. licheniformis* DSM13 was used and shown to be successfully expressed in all vectors (Figure 3B). This study confirms that these vectors can be used for secreted expression in *B. subtilis*. As such, the vectors could be explored for the replacement of native leader sequences with known sequences, particularly useful when the native enzyme sequence is divergent and may escape recognition by the *Bacillus* secretion systems.

Comparison to protein yields in *B. megaterium*, for which the vector backbones were optimized, is outside the scope of this study. In the aforementioned leader sequence screen, however, it was found that the YocH leader sequence promoted higher yields of exported target enzyme than the LipA sequence [27]. In our study, based on expression and activity levels of recombinant subtilisin from *B. licheniformis* DSM13, it was not possible to discriminate between results obtained with native and artificial leader sequences (Figure 3D). Induction at shorter intervals (1.5 h) did not change the results. To identify the optimal leader sequence, empirical testing on a higher number of recombinant proteins may be required, but the fact that results vary between reports demonstrates the merit of including several construct designs in a screen at this time point.

The *Bacillus* system has been assessed by expressing four homologous subtilisins that are divergent in sequence and expected to have different temperature activity profiles [16]. These were previously expressed in an active form in *E. coli*. Using our p17 vector and exploring the native leader sequences for export, all subtilisins were identified as soluble and active in the growth medium (Figure 4B). These subtilisins used in the mini screen are all alkaline, as confirmed by their activity at high pH (Figure 4D). They differ somewhat in the activity profiles, with the *B. subtili*s homolog having a lower optimal temperature than the *B. licheniformis* and *B. paralicheniformis* homologs. Apparently, the latter two homologs share similar activity profiles. This can be explained by a 98% sequence identity according to a pairwise sequence alignment [16]. Moreover, they share a profile with the commercial Alcalase™, which also originates from *B. licheniformis*. The *B. amyloliquefaciens* homolog has an optimal activity at 50 °C, which aligns well with other reports [43], but appears to have a broader temperature optimum range than the other homologs. This trait could be useful in industrial applications to avoid the enzyme activity from dropping due to temperature fluctuation. Apparently, most activity is lost at 80 °C which aligns with industrial conditions for enzyme inactivation that commonly occur at 90 °C.

## 5. Conclusions

To conclude, the *Bacillus* system herein reported complements the previously developed *E. coli* system [16], and allows rapid switching between two commonly used heterologous host systems for comparative expression. Moreover, we show that the vectors described are not limited to the expression of certain enzymes, here exemplified by the expression of both subtilisins and green fluorescent protein, but could also be applied to other enzymes for more generalized enzyme discovery or development.

## Supplementary Materials

The following are available online at http://www.mdpi.com/2076-2607/6/2/51/s1, Table S1: Plasmids and strains used in this study; Table S2: Primers used in this study.
