# Supplementary material for: Development of Versatile Vectors for Heterologous Expression in Bacillus
Source: Microorganisms. 2018 Jun 7;6(2):51. doi: 10.3390/microorganisms6020051 (PMC6027494; doi:10.3390/microorganisms6020051)
Supplement: Supplementary file 1 [file microorganisms-06-00051-s001.pdf]

microorganisms

Supplementary data

Table S1. Plasmids and strains used in this study.

Plasmid name	Application	Resistance marker[1]	Fusion partner	Promoter	Reference /Source
pINITIAL	Cloning	cam	-	-	[23]
p1	Expression	amp	N-his	*pBAD*	[16,23]
pSP_{LipA}-hp	Expression	amp, tet	SP_{LipA}, C-his	*pXylA*	MoBiTec
pSP_{YocH}-hp	Expression	amp, tet	SP_{YocH}, C-his	*pXylA*	MoBiTec
pSSBm85	Expression	amp, tet	-	*pXylA*	[27]
pUC57_SapI-free.kan	Cloning	kan	-	-	GenScript
p17	Expression	amp, tet	C-his	*pXylA*	This study
p18	Expression	amp, tet	SP_{LipA}, C-his	*pXylA*	This study
p19	Expression	amp, tet	SP_{YocH}, C-his	*pXylA*	This study

Strain	Properties	Source
Escherichia coli MC1061	General cloning and expression from *pBAD* promoter, streptomycin resistant	[23]
E. coli DB3.1	*ccd*B resistant, streptomycin resistant	Invitrogen
Bacillus subtilis WB800N	Eight protease deficient, neomycin resistant	Mobitec

[1] Cam, chloramphenicol; amp, ampicillin; tet, tetracyclin; kan, kanamycin

Table S2. Primers used in this study. .

Primer name	Sequence (5'-3')	Application
p17_ccdB_F	TGTTCACTTAAATCAAGGAGGTGAATGTACAATGAG TAGAAGAGCGAGCTGCA	Cloning
p18_ccdB_F	CGTCTGGCGCAGGCGCCGCAAGTAGAAGAGCGAGCT GCA	Cloning
p19_ccdB_F	GGCAAGTGGTGCATCAGCTGCAAGTAGAAGAGCGA GCTGCA	Cloning
pSP_ccdB-R	ACCGGTTAGTGATGGTGATGGTGATGTGCAGAAGAG CTGAACTAGTG	Cloning
pSP_SQ-F	GAGATAAAGTTAGTTTATTGG	PCR screening
pSP_SQ-R	GATGGATATGTTCTGCC	PCR screening
91_SQccdB_R	GAAAATGACATCAAAAACGCCATTAACC	Sequencing
251_SQFXcat_F	CATTTTACGTTTCTCGTTCAGCTTTTTG	Sequencing
